# An Advanced Deep Learning Approach for Ki-67 Stained Hotspot Detection and Proliferation Rate Scoring for Prognostic Evaluation of Breast Cancer

**DOI:** 10.1038/s41598-017-03405-5

**Published:** 2017-06-12

**Authors:** Monjoy Saha, Chandan Chakraborty, Indu Arun, Rosina Ahmed, Sanjoy Chatterjee

**Affiliations:** 1 School of Medical Science and Technology, Indian Institute of Technology, Kharagpur, West Bengal India; 2 Tata Medical Center, New Town, Kolkata, West Bengal India

## Abstract

Being a non-histone protein, Ki-67 is one of the essential biomarkers for the immunohistochemical assessment of proliferation rate in breast cancer screening and grading. The Ki-67 signature is always sensitive to radiotherapy and chemotherapy. Due to random morphological, color and intensity variations of cell nuclei (immunopositive and immunonegative), manual/subjective assessment of Ki-67 scoring is error-prone and time-consuming. Hence, several machine learning approaches have been reported; nevertheless, none of them had worked on deep learning based hotspots detection and proliferation scoring. In this article, we suggest an advanced deep learning model for computerized recognition of candidate hotspots and subsequent proliferation rate scoring by quantifying Ki-67 appearance in breast cancer immunohistochemical images. Unlike existing Ki-67 scoring techniques, our methodology uses Gamma mixture model (GMM) with Expectation-Maximization for seed point detection and patch selection and deep learning, comprises with decision layer, for hotspots detection and proliferation scoring. Experimental results provide 93% precision, 0.88% recall and 0.91% F-score value. The model performance has also been compared with the pathologists’ manual annotations and recently published articles. In future, the proposed deep learning framework will be highly reliable and beneficial to the junior and senior pathologists for fast and efficient Ki-67 scoring.

## Introduction

Automated breast cancer (BC) detection research has been increased nowadays due to the inflation of BC mortality rate worldwide^1^. In the GLOBOCAN 2012, BC has been reported as the second most common cancer, which occurs mostly among women than men^2, 3^. As per the Nottingham grading system, BC grading is done based on the scores of nuclear pleomorphism, mitotic count, and tubule formation^4^. Additionally, to confirm the BC subtypes, to distinguish normal and malignant tumor and to guide treatment decisions smoothly, immunohistochemical (IHC) analysis of breast tissue is required. The most commonly used IHC markers are Ki-67, estrogen receptors, progesterone receptor, protein P53 and human epidermal growth factor-2^5^.

**Figure 1:**
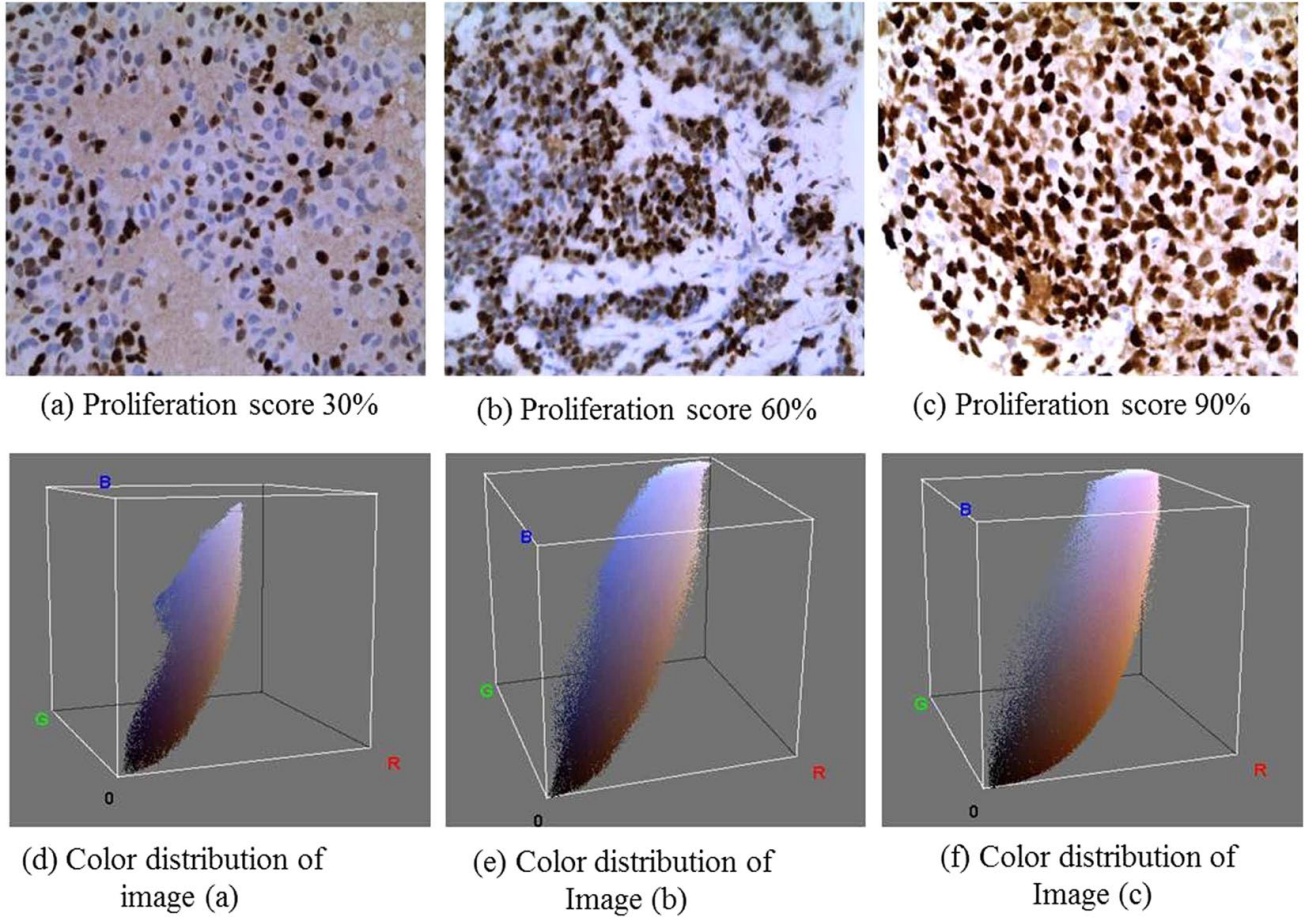
Ki-67 proliferation scoring by the pathologists with respect to differential color distribution: Three input Ki-67 stained images of breast cancer at 40× with the scores (**a**) = 30%; (**b**) = 60%; (**c**) = 90%; and color spectrum visualization of the inputs images (**d–f**).

Ki-67, non-histone protein, is one of the essential prognostic and predictive markers for BC detection. Gerdes *et al*.^6, 7^ reported that Ki-67 signature is exhibit only in proliferating cells and disappears in quiescent cells. Furthermore, the Ki-67 expression doesn’t appear in G0 cell cycle but instead appears in G1, S, G2, M cell cycle^7^. The level of Ki-67 becomes low during G1 and S cell cycle phase but increases during mitosis (exception anaphase and telophase). Mitotic index is considered as one of the most significant proliferation markers for BC grading or screening. Although it has some limitations, e.g., the rate of mitosis proliferation is non-linearly related to the number of mitosis in high power field^8^. Hence, the IHC analysis of Ki-67 using monoclonal antibody has emerged for the alternative assessment for the proliferation index^9^. The proliferation score determines the severity of BC as follows: low (<15%), average (16–30%) and highly (>31%) proliferate^5^. Patients with high Ki-67 is very sensitive to radiotherapy and chemotherapy^10^. Ki-67 expression possesses the significant predictive and prognostic value in BC. Personalized treatment and diagnosis facility can improve the survival rates of BC patients. Hence, the identification of accurate grading (grade I, grade II and grade III) remains always a challenge for pathologists. Till now, the clinical decision on BC grading is mostly made manually based on both predictive and prognostic pathological markers. The manual assessment of Ki-67 is subjective, error-prone and dependent on the Intra and inter-observer ambiguities. Moreover, in the rural and urban areas with minimum or few advanced instrumentation, manual inspection of Ki-67 scoring may provide wrong results. Henceforth, automated assessment of Ki-67 scoring is highly required. The automatic scoring will provide high throughput, more objective and reproducible results in comparison with the manual evaluation. The proliferation score is calculated as the ratio between total numbers of immunopositive nuclei and a total number of nuclei present in the image^11^. The immunopositive (brown color) and immunonegative (blue color) nuclei together called as hotspots. Figure 1 shows the Ki-67 stained BC images according to their proliferation score and their corresponding color distribution map using open source ImageJ software. To date, the Ki-67 automated assessment was done mainly based on conventional imaging techniques. But due to the heterogeneous and massive dataset in medical imaging or biomedical applications scientists are getting interested in deep learning. Deep learning is a versatile biomedical research tool with numerous potential applications. P. Mamoshina *et al*. (2016) proposed a deep learning framework in biomedicine application^12^. Y. Xu *et al*. (2014) reported deep learning for medical image analysis^13^. This paper has been structured as an introduction, literature review, experimental setup, results & discussion and finally conclusion.

### Literature review

Many machine learning techniques have been published for Ki-67 scoring using IHC stained BC images. To the best of our knowledge, most of the Ki-67 scoring methods are based on conventional machine learning techniques. Based on the extensive literature survey, we conclude that there are no such reports available till date specially aimed to the deep learning approaches for considering much finer information inherent in the microscopic images. Table 1 shows the characterization of different Ki-67 scoring methods.

**Table 1:** Characterization of Ki-67 scoring approaches.

Categories	Year	Cancer type	Methodology used	Results
*Conventional techniques*	2016	Nasopharyngeal cancer	K-means clustering	91.8% Segmentation accuracy^15^
	2015	Meningiomas and Oligodendrogliomas tumor	Morphology operation, thresholding, feature extraction and classification	The results shows the effectiveness of the proposed algorithm^33^
	2014	Neuroendocrine tumor	Learning based approach	89% precision, 91% recall, 90% F-score^11^
	2014	Breast Cancer	Otsu thresholding	High correlation observed between manual and automated procedure^34^
	2014	Breast cancer	Aperio Genie and Nuclear v9 software	Misclassification rate 5–7%^35^
	2013	Pancreatic neuroendocrine tumor	Voting-Based Seed Detection, Repulsive Deformable Model, Two step classification	87.68% classification accuracy, 88.01% sensitivity and 87.12% specificity^36^
	2013	Rabbit Liver	Inform 1.4 image analysis software	Useful in clinical practice^37^
	2012	Breast cancer	K-means clustering	T-test shows reliable proliferation rate^38^
	2012	Not mentioned	Watershed segmentation, Laplacian-of-Gaussian filtering, SVM classifier	90% sensitivity at confidence level I, 99% sensitivity at confidence level VIII^39^
	2012	Breast Cancer	Slidepath Tissue IA system software	Excellent agreement between manual and automated technique^40^
	2010	Breast cancer	ImmunoRatio software	20% labeling index as a cutoff, 2.2 hazard ratio^41^
	2009	Meningiomas tumor	Thresholding, watershed and morphological operations, SVM classifier	The proposed method helpful for further research^42^
*Deep Learning*	*No work has been carried out for Ki-67 scoring using deep learning approaches*			

#### Review on conventional techniques

M. Abubakar *et al*.^14^ proposed a computer vision algorithm for Ki-67 scoring in BC tissue microarray images. Their algorithm shows promising performance measure in comparison with other scoring technique. The authors achieved 90% classification accuracy with 0.64 kappa value. The automated quantification of Ki-67 using nasopharyngeal carcinoma has been described in P. Shi *et al*.^15^. Their algorithm mainly consists of smoothing, decomposition, feature extraction, K-means clustering, and quantification. They achieve 91.8% segmentation accuracy. F. Zhong *et al*. (2016) compared the visual and automated Ki-67 scoring on BC images^16^. The authors used total 155 Ki-67 immunostained slides of invasive BC. The scoring has been performed based on hotspots and average score. They employed correlation coefficient to analyze the consistency and to minimize the errors between the two techniques. Z. Swiderska *et al*.^17^ employed computer vision algorithm for hotspots selection from the whole slide meningioma images. The authors used color channel selection, Otsu thresholding, morphological filtering, feature selection and classification. The authors achieved high correlation between manual and automated hotspots detection. F. Xing *et al*. (2014) proposed an automated machine learning algorithm for Ki-67 counting and scoring using neuroendocrine tumor images^11^. The proposed algorithm has three stages. Stage-I comprises of seed point detection, segmentation, feature extraction and cell level probability measurement. Stage-II consists of tumor or non-tumor cell classification, probability map generation, and feature extraction. Finally, stage-III provide immunopositive and negative cell classification along with Ki-67 scoring. This approach achieved 89% precision, 91% recall and 90% F-score. J. Konsti *et al*.^18^ reported virtual application for Ki-67 assessment in BC. The algorithm mainly developed using ImageJ software. At first, images were processed using color deconvolution to separate hematoxylin and diaminobenzidine stain color channels. Then a mask was moved over the images to get target objects. This approach showed 87% agreement and 0.57 *kappa* value. The Ki-67 scoring using Gamma-Gaussian Mixture Model (GGMM) has not been attempted yet. Khan *et al*. (2012) proposed GMM model for mitosis identification from histopathological images^19^.

#### Review on deep learning techniques

To the best of our knowledge, automatic Ki-67 scoring and hotspots detection using deep learning approach were not attempted yet.

The major contributions of this paper include:

- Development of an advanced deep learning model for Ki-67 stained hotspots detection and calculation of proliferation index.
- Inclusion of decision layer in the proposed deep learning framework.
- It is a value addition in terms of main quantification in the already existing established techniques for Ki-67 scoring.

### Experimental setup

#### Slide preparation and image acquisition

The slide having histological sections of the tissue biopsy was stained by using Ki-67 monoclonal antibody. At 40x magnification, total 450 microscopic images from 90 (histologically confirmed) slides were grabbed and digitally stored using Zeiss Axio Imager M2 microscope with Axiocam ICc5 camera at constant contrast and brightness in BioMedical Imaging Informatics (BMI) Lab of School of Medical Science & Technology, IIT Kharagpur and Department of pathology, Tata Medical Center (TMC), Kolkata. The field of view (FOV) of the each image-matrix was 2048 × 1536 pixels (width × height). All the images contained almost 259,884 (131,053 *immunopositive* and 128,831 *immunonegative*) annotated and un-annotated nuclei. All procedures, e.g. slide preparation, image acquisition, etc. were performed in accordance with the institutional guidelines. The ethical statement details have been discussed in Ethics and consent statements.

#### Layers of Convolutional Network (CN)

A CN mainly consists of multiple consecutive convolution layers, subsampling/pooling layers, non-linear layers and fully-connected layers. Let, *f* is a CN and a composition of a sequence of *N* number of layers or functions (*f*_1_, *f*_2_…*f*_*N*_). The mapping between input (*w*) and output (*u*) vector of a CN can be represented as^20^:

1 $$u=f(w;{X}_{1},{X}_{2},{X}_{3},\ldots ,{X}_{N})={f}_{1}(w;{X}_{1})\circ {f}_{2}(.;{X}_{2})\ldots \circ {f}_{N-1}(.;{X}_{N-1})\circ {f}_{N}(.;{X}_{N})$$

Conventionally, *f*_*N*_ has been assigned to perform convolution or, non-linear activation or, spatial pooling. Where *X*_*N*_ denotes the bias and weight vector for the *N* ^th^ layer *f*_*N*_. Given a set of *η* training data ${\{({w}^{(i)},{u}^{(i)})\}}_{i=1}^{\eta }$, we can estimate the vectors (*X*_1_, *X*_2_, *X*_3_, …, *X*_*N*_) as follows

2 $$\mathop{\text{arg}\,\min }\limits_{{X}_{1},{X}_{2},{X}_{3},\ldots ,{X}_{N}}\frac{1}{\eta }{\sum }_{i=1}^{\eta }\, {f}_{Loss}(f({w}^{(i)};{X}_{1},{X}_{2},{X}_{3},\ldots ,{X}_{N}),{u}^{(i)})$$

where *f*_*LOSS*_ indicates loss function. The equation 2 can be performed using stochastic gradient descent and backpropagation methods.

#### Convolution layers

In deep learning, the convolution operation extracts different low-level (e.g. lines, edges, and corner) and higher-level hierarchical features from the input images. In our proposed deep learning framework multiple layers are stacked in a way so that the input of *h* ^th^ layer will be the output of (*h*−1)^th^ layer. A convolutional layer usually learns convolutional filters to calculate feature map. The equation^21^ of feature map ($F{M}_{m}^{h}$) at a level *m* will be

3 $$F{M}_{m}^{h}=f({\alpha }_{m}^{h}+\sum _{j}F{M}_{j}^{h-1}\times {G}_{jm}^{h})$$

In equation 3, $j\in [0,\sum _{j}F{M}_{in}^{h-1}-1]$ represents input feature map indices and $m\in [0,\sum _{j}F{M}_{out}^{h}-1]$ denotes output feature map indices. Here, $F{M}_{in}^{h-1}$ and $F{M}_{out}^{h}$ represent a number of input and output feature maps at *h* ^th^ level. ${G}_{jm}^{h}$ and ${\alpha }_{m}^{h}$ represent biases and corresponding kernels respectively. In each convolution layer, there are two components which create feature maps. The first element is Local Receptive Field and the second part is shared weights. A feature map is the output of one filter applied to the previous layer. The each unit in a feature map looks for the same feature but at different positions of the input image.

#### Max-pooling layer

The pooling layers have been employed to get spatial invariance by reducing the feature maps’ resolution. The pooling operation makes the features more robust against distortion and noise. There are two types of pooling mostly used in deep learning. Those are average pooling and max-pooling. In both the cases, the input is divided into two-dimensional spaces (non-overlapping). Based on our requirement and image characteristics we have chosen max-pooling operations in our proposed framework. The advantages of this type of layer are the capability of downsampling the input image size and create positive invariance over the local regions. The max-pooling function has been calculated using the below equation^22, 23^.

4 $${{\rm{\Psi }}}_{j}=\,\max ({\psi }_{i}^{n\times n}z(n,n))$$

The max-pooling window can be overlapped and arbitrary size. Here *ψ* is the input image, *z* denotes window function and *n* × *n* = 71 × 71 is the input patch size.

#### Rectified Linear Unit

In deep learning, Rectified Linear Units (ReLUs) have been used as an activation function and as a gradient descent vector. It is defined by the below equation^24, 25^

5 $$q(r)=\,\max (0,r)$$

where *q* denotes model’s output function with an input *r*. The size of input and output of this layer is same. The ReLU enhances the performance of the network without disturbing receptive fields and increases nonlinearity of the decision function. ReLU trains the CN much faster than the other existing non-linear functions (e.g., sigmoid, hyperbolic tangent and absolute of hyperbolic tangent).

#### Fully Connected (FC) Layer

The FC layer is often used as a final layer of a CN in a classification problem. This layer mathematically sums a weighting of features of a previous layer. It works like a classifier. This layer is not spatially located and serves as a simple vector. In the proposed model, FC layer height and width of each blob is set to 1.

#### Dropout Layer

Dropout is a regularization technique which is mostly used for reducing overfitting and preventing complex-co-adaptions on training data. Due to this layer, the learned weights of nodes become more insensitive to the weights of the other nodes. This layer helps to increase the accuracy of the model by switching off the unnecessary nodes in the existing network. The dropout neurons do not contribute in the backpropagation and forward pass.

#### Decision Layer

An additional decision layer comprises of decision trees, has been introduced in the proposed deep learning framework. As per our knowledge, the concept of decision layer in deep learning framework has not been used so far. The inclusion of decision layer increases the performance of the proposed model. In our proposed method we used decision trees inspired by P. Kontschieder *et al*.^26^. The proposed decision layer consists of decision nodes and prediction nodes. The decision layer algorithm is a recursive algorithm and implemented in C++^27^. The column of the blob data table has been split based on information gain or least entropy.

Let, the input and finite output spaces are denoted by *X* and *Y* respectively. In the decision tree, decision nodes are also called internal nodes of the tree and indexed by *D*. Similarly, prediction nodes are called terminal nodes and indicated by *P*. Each decision node *0* ∈ *D* assigned a decision function ${f}_{d}(.;{\rm{\Theta }}):X\to [0,1]$. Each projection node *p* ∈ *P* possesses probability distribution *π*_*p*_ over *Y*. When a sample *x* ∈ *X* reaches a decision node *d* it will send to the right or left subtree based on the output of ${f}_{d}(x;{\rm{\Theta }})$. On decision trees *f*_*d*_ are binary, and the routine is deterministic. The final prediction result for sample *x* from tree *T* with decision notes parametrized by Θ is denoted by

6 $${P}_{T}[y|x,{\rm{\Theta }},\pi ]={\sum }_{p\in P}{\pi }_{py}{\mu }_{p}(x|{\rm{\Theta }})$$

Here, *π*_*py*_ and *π* = (*π*_*p*_)*p* ∈ *P* represents the probability of a sample reaching leaf *p* on class *y* and routine function indicated by ${\mu }_{p}(x|{\rm{\Theta }})$. When *x* ∈ *X*, $\sum _{p}{\mu }_{p}(x|{\rm{\Theta }})=1$.

In decision nodes decision function works based on stochastic routine and is defined as

7 $${f}_{d}(x;{\rm{\Theta }})=\sigma ({f}_{r}(x;{\rm{\Theta }}))$$

Here *σ*(*x*) is a sigmoid function and defined as $\sigma (x)=\frac{1}{(1+{e}^{-x})}$. The ${f}_{r}(.;{\rm{\Theta }}):X\to {\mathbb{R}}$ is a real-valued function. An ensemble of decision trees are called decision forest and are denoted by

8 $$F=\{{T}_{1},{T}_{2},\ldots ,{T}_{z}\}$$

**Figure 2:**
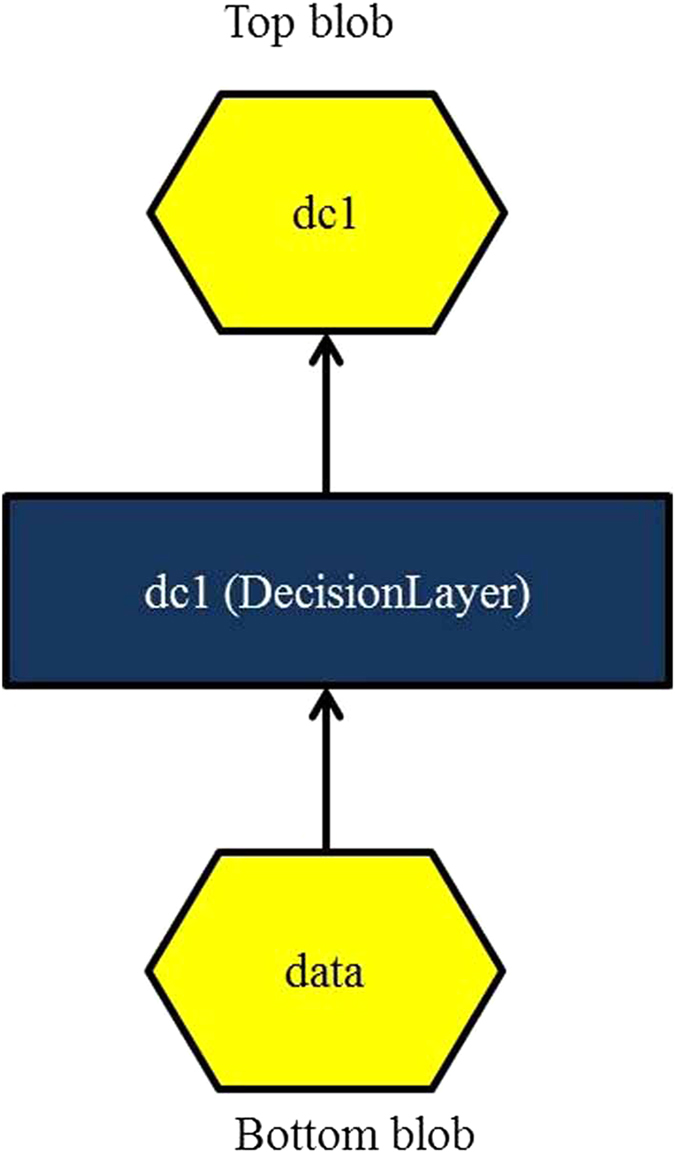
Shows pictorial representation of decision layer connections in CAFFE.

The learning of decision trees along with decision nodes and prediction nodes have been done using CAFFE stochastic gradient descent approach. The pictorial representation of decision layer connections in CAFFE has been shown in Fig. 2.

#### Patch selection

**Figure 3:**
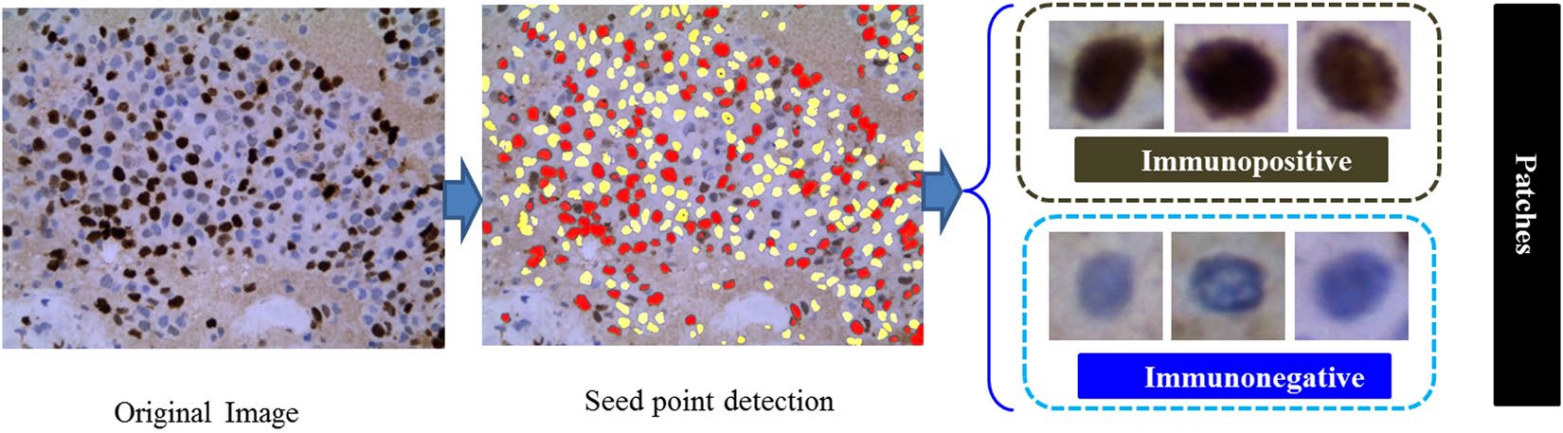
Illustrates the flow diagram of the patch detection from original images.

Patch selection is a very much essential part of the proposed methodology. The overall patch selection work flow diagram has been shown in Fig. 3. Due to variations of nuclei size, shape and the localization of nuclei, image patches may vary. In the case of overlapping nuclei, it is very complicated to crop a patch which will only contain a single nucleus (immunopositive or immunonegative). Henceforth, we have detected seed point using Gamma mixture model (GMM) with Expectation-Maximization algorithm. The algorithm is an iterative method and used to find maximum posterior or maximum likelihood. The iteration alternates between performing an expectation (E) and maximization (M) for each parameter.

Let *I* is an image *I* = (*I*_1_, *I*_2_, *I*_3_, …, *I*_*Q*_) where *Q* represents a number of pixels and *I*_*Q*_ denotes gray-level intensity of a pixel. To infer a configuration of positive labels *L*, *K* = (*K*_1_, *K*_2_, *K*_3_, … *K*_*Q*_) where *K*_*Q*_ ∈ *L L* = {0, 1}. Now as per MAP criteria the labeling satisfies:

9 $${K}^{\ast }=\mathop{\text{arg}\,\max }\limits_{k}\{Y(I|K,{\rm{\Theta }})Y(K)\}$$

Here, *Y*(*K*) is a Gibbs distribution. In the Expectation-Maximization algorithm the equation 9 can be written as

10 $${K}^{\ast }=\mathop{\text{arg}\,\max }\limits_{K\in k}\{U(I|K,{\rm{\Theta }})+U(K)\}$$

Here, *U* denotes urinary potential or likelihood energy and denoted by

11 $$U(I|K,{\rm{\Theta }})={\sum }_{Q}[\frac{{({I}_{Q}-{\mu }_{KQ})}^{2}}{2{\sigma }_{K}^{2}}+\,\mathrm{ln}\,{\sigma }_{K}]$$

As per the hypothesis, we are assuming that the segmented region’s intensity will follow a Gaussian distribution with parameters *σ*_*xi*_ = (*μ*_*xi*_, *σ*_*xi*_). This hypothesis is unable to model real-life objects. So for complex distribution GMM is the best choice for the engineers. A GMM with *c* components is represented by below equations^28^:

12 $${\sigma }_{i}=\{({\mu }_{i,1},{\sigma }_{i,1},{w}_{i,1}),\ldots ,({\mu }_{i,c},{\sigma }_{i,c},{w}_{i,c})$$

The Gaussian distribution with parameters can be written as

13 $$G(z;{\alpha }_{i})=\frac{1}{\sqrt{2\pi {\sigma }_{i}^{2}}}\exp (-\frac{{(z-{\mu }_{i})}^{2}}{2{\sigma }_{i}^{2}})$$

Comparing the equation 12 with equation 13, we get the weighted probability as follows

14 $${G}_{mix}(z;{\alpha }_{i})={\sum }_{c=1}^{h}{w}_{i,c}G(z;{\mu }_{i,c},{\sigma }_{i,c})$$

For a color RGB image the pixel intensity is a 3-dimensional vector. The parameters of GMM now becomes^28^

15 $${\alpha }_{xi}=({\mu }_{i,1},{\sum }_{i,1},\,{w}_{i,1})\ldots ({\mu }_{i,c},{\sum }_{i,c},\,{w}_{i,c})$$

Comparing equation 15 with equation 11, we will get the likelihood Energy Equation as below

16 $$U(I|K,{\rm{\Theta }})={\sum }_{Q}[\frac{1}{2}{({I}_{Q}-{\mu }_{KQ})}^{T}{\sum }_{KQ}^{-1}({I}_{Q}-{\mu }_{KQ})+\,\mathrm{ln}|{\sum }_{KQ}|\frac{1}{2}]$$

The seed points of immunopositive and immunonegative nuclei have been denoted as red and yellow color respectively. Finally, each patch of size 71 × 71 have been cropped using centroid points of each selected seed points, and lastly, patches have been feed into our proposed deep learning framework.

### Proposed Deep Learning Model (DLM)

**Figure 4:**
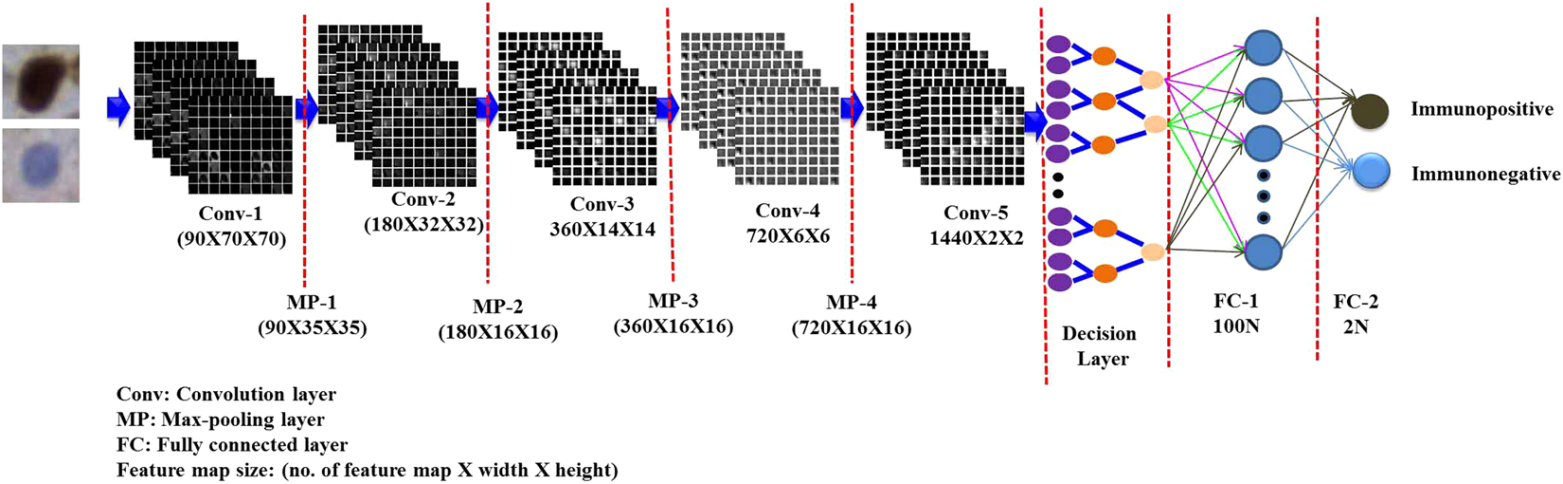
Shows the flow diagram of the proposed deep learning model.

The proposed DLM has been developed using CAFFE deep learning framework, and CUDA enabled parallel computing platform^29^. The architectural details of the proposed DLM have been shown in Table 2. The proposed model includes one decision layer, two fully connected layers, four max-pooling layers, five convolution layers and six ReLUs. The decision layer has been added after fifth convolutional layer. ReLU has been employed after each convolutional layer to fasten the computing time. Dropout layer has been inserted after first FC layer to avoid the over-fitting. After the rigorous experiment, it was found that dropout ratio = 0.5 is provided the best result in this dataset. The workflow diagram of the proposed DLM has been illustrated in Fig. 4. Our proposed model learns from the labeled data.

**Table 2:** Proposed deep learning approach.

Layer	Type	Maps	Neurons	Filter size
0	Input Image	3	71 × 71	—
1	Conv-1	90	70 × 70	2 × 2
2	MP-1	90	35 × 35	2 × 2
3	Conv-2	180	32 × 32	4 × 4
4	MP-2	180	16 × 16	2 × 2
5	Conv-3	360	14 × 14	3 × 3
6	MP-3	360	7 × 7	2 × 2
7	Conv-4	720	6 × 6	2 × 2
8	MP-4	720	3 × 3	2 × 2
9	Conv-5	1440	2 × 2	2 × 2
10	*Decision layer*	—	720	1 × 1
11	FC-1	—	100	1 × 1
12	FC-2	—	2	1 × 1

#### Parameter initialization

The numbers of training and validation samples were considered as 70% and 30% respectively out of 450 images. The training and validation batch size were set to 128. The testing interval and maximum iteration were assigned to 5000 and 450,000 respectively. The other important parameters include learning rate (=0.01), weight decay (=0.005) and momentum (=0.85). The detailed source code of the model and parameter initialization files has been included as supplementary documents.

#### Ethics and consent statements

Ethical approval has been taken from the TMC, Kolkata (ref. no. EC/GOVT/07/14; dated August 11, 2014) and Indian Institute of Technology, Kharagpur (ref. IIT/SRIC/SAO/2015; dated July 23, 2015) to conduct this research work. The patient consent forms have been signed by the patient and their close relatives. The slides were prepared and maintained by TMC, Kolkata. All procedures, e.g. slide preparation, image acquisition, etc. were performed in accordance with the institutional policies.

## Results and Discussion

In this portion, we assess the efficacy of our proposed deep learning framework. We randomly divided the image patch dataset into five subsets (5-fold cross validation); each subset includes 20% of the total data. It should be noted that during training phase each time we performed patch selection, model learning and classification using the four subsets. Finally, the selected patches and trained model were used to assess the performance of the left-out testing sub-dataset. Five-fold cross validation results have been shown in Table 3. Furthermore, performance based on various combinations of training and testing dataset has been indicated in Table 4. The seed point selection and object detection algorithms have been developed using MATLAB and Python tools on a machine with AMD Opteron processor 128 GB RAM, NVIDIA Titan X pascal GPU. The proposed cascaded framework achieved almost 0.974 training accuracy and 0.0945 loss.

**Table 3:** 5-fold cross-validation.

Cross-Validation	Pr	Re	F-score
1^st^	0.930	0.881	0.910
2^nd^	0.927	0.875	0.910
3^rd^	0.926	0.879	0.920
4^th^	0.930	0.881	0.900
5^th^	0.931	0.880	0.910
Average	**0.930**	**0.880**	**0.910**

**Table 4:** Performance based on various combinations of training and testing dataset.

Training images (%)	Testing images (%)	Pr	Re	F-score
**0**	**100**	0.909	0.777	0.838
**25**	**75**	0.925	0.877	0.901
**50**	**50**	0.929	0.880	0.904
**75**	**25**	0.950	0.882	0.914
**100**	**0**	0.971	0.893	0.930

### Quantitative evaluation

**Figure 5:**
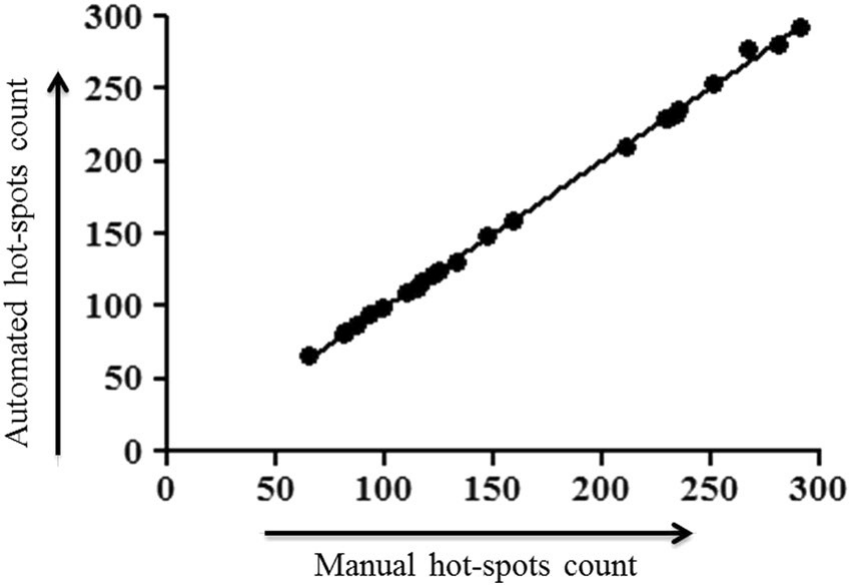
Regression curve between automated and manual hotspots count.

The quantitative assessment results have been shown in Table 5. Figure 5 illustrates the regression curve (R^2^ = 0.9991) between automatic and manual hotspots detection. The graph indicates that the model generated immunopositive and immunonegative nuclei count provides almost exact results in comparison to the pathologists’ count. The model is evaluated using precision (Pr), recall (Re) and F-score as below^30^:

17 $$\Pr \, ( \% )=\frac{{\rm{True}}\,{\rm{positive}}}{{\rm{True}}\,\mathrm{positive}+\mathrm{False}\,{\rm{negative}}}\times 100$$

18 $$\mathrm{Re}\, ( \% )=\frac{{\rm{True}}\,{\rm{positive}}}{{\rm{True}}\,\mathrm{positive}+\mathrm{False}\,{\rm{positive}}}\times 100$$

19 $$F\,( \% )=2\times (\frac{\Pr \times \mathrm{Re}}{\Pr +\mathrm{Re}})\times 100$$

**Table 5:** Quantitative performance measures for Ki-67 scoring.

Confusion Matrix		Pr	Re	F-score
17028	2277	0.93	0.88	0.91
1287	15840

**Figure 6:**
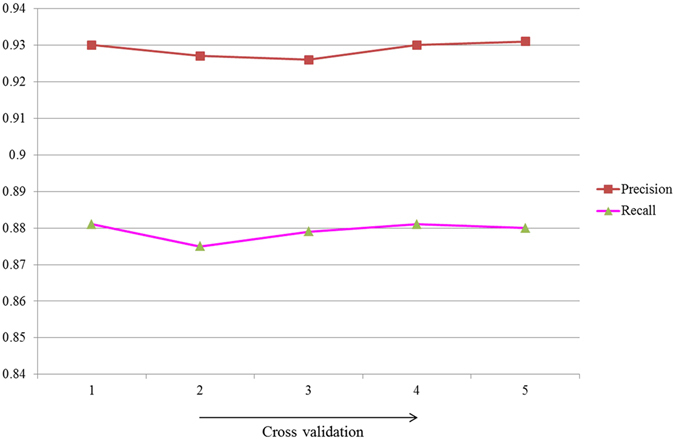
Shows precision and recall curve.

Our proposed model achieved 93% precision, 88% recall and 91% F-score value. We also added confusion matrix for better understanding the results. Figure 6 shows precision and recall curve across 5-fold cross-validation.

### Qualitative evaluation

**Figure 7:**
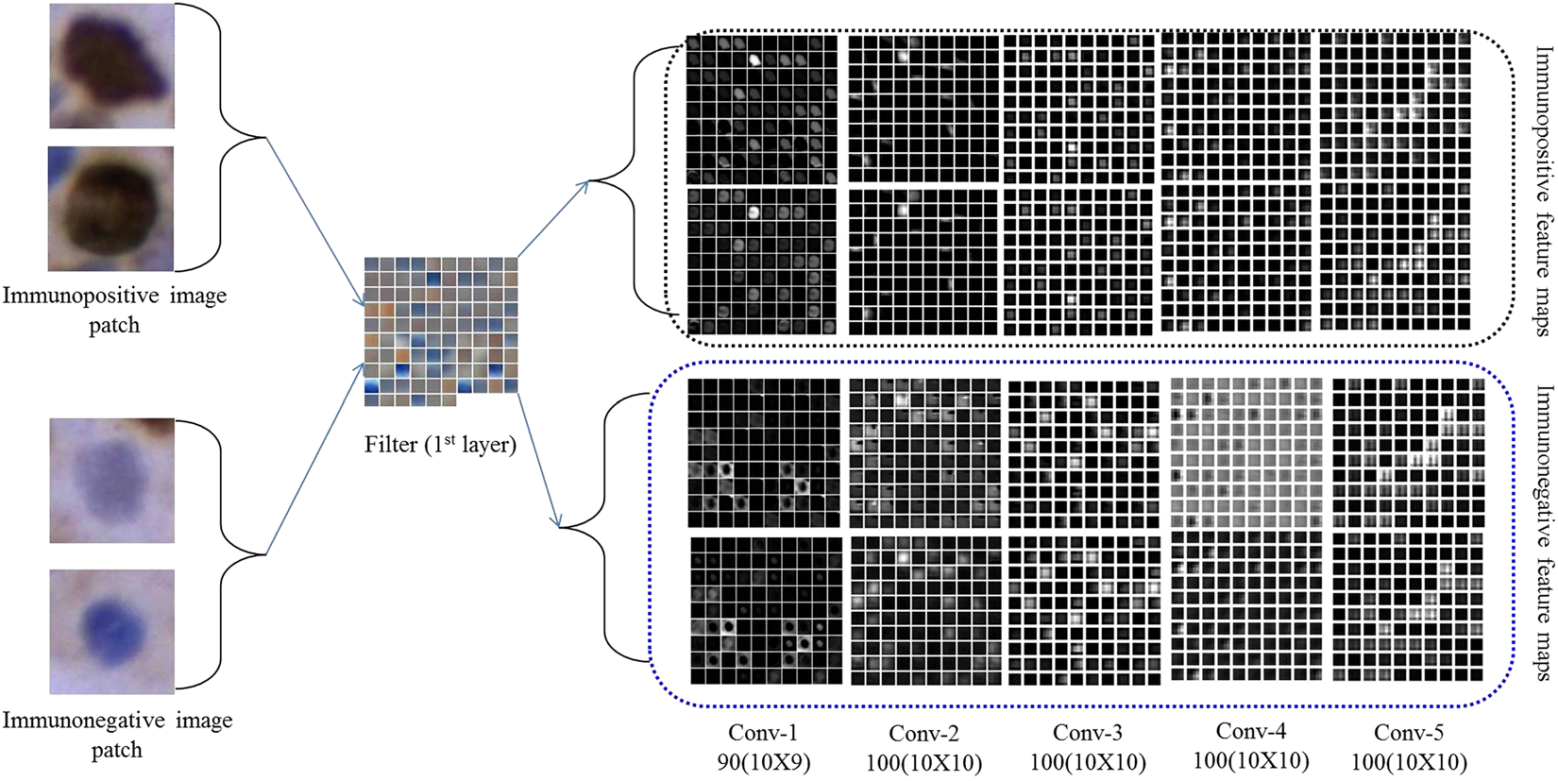
Visualization of feature maps of various convolution layers.

**Figure 8:**
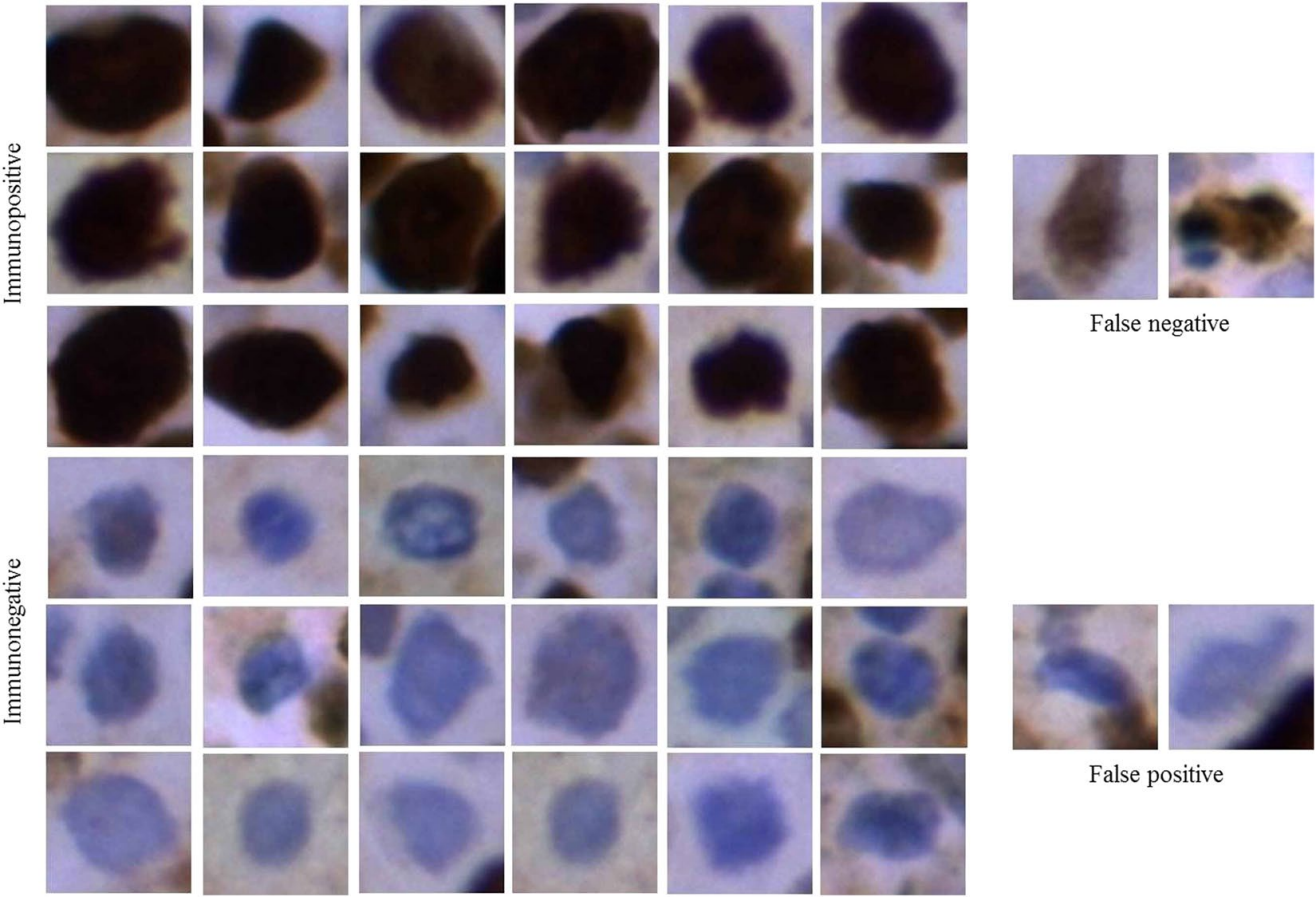
Ki-67 detection results by using the proposed algorithm.

**Figure 9:**
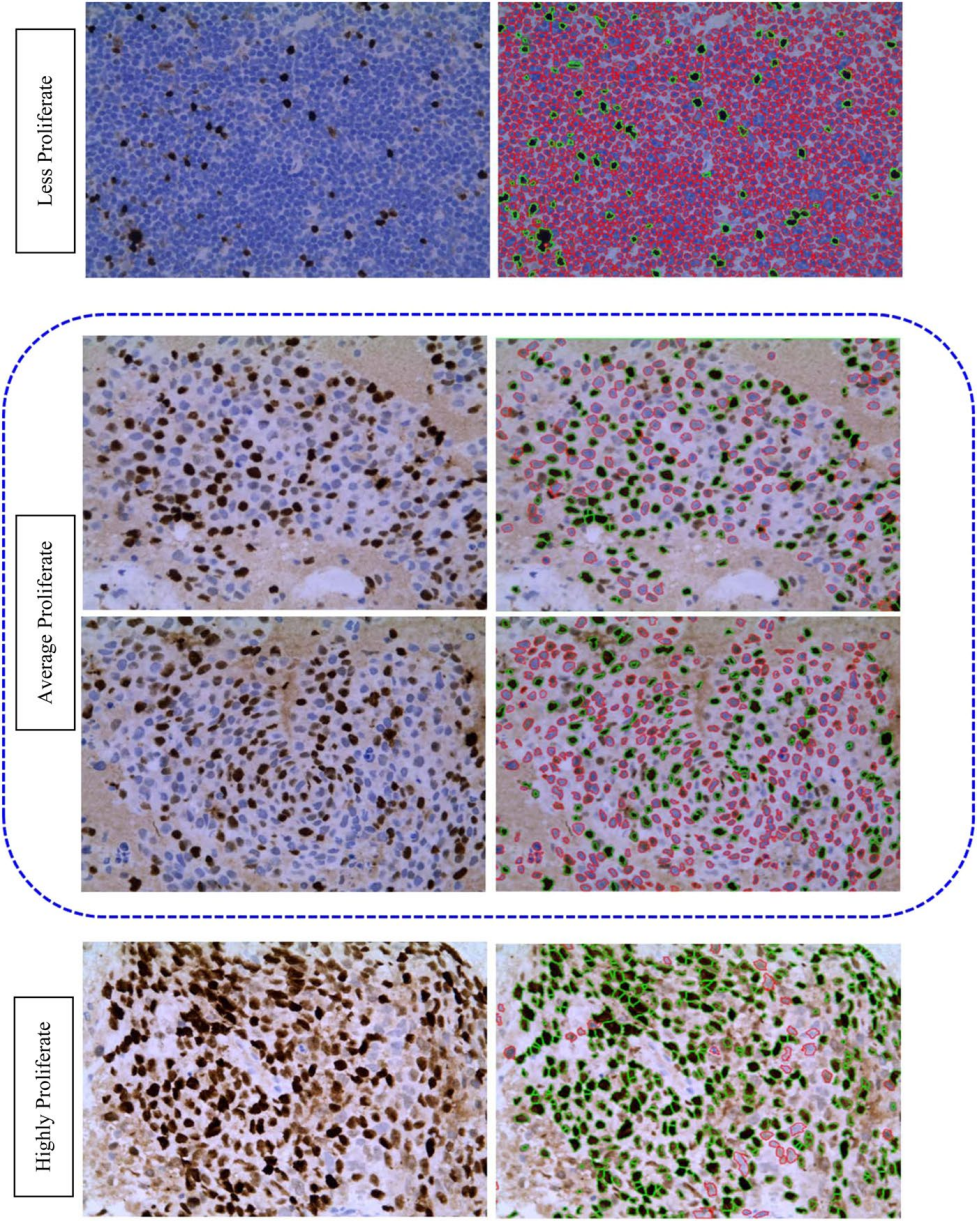
Overall detection of hotspots in breast cancer IHC images at different proliferation levels.

The first, second, third, fourth and fifth convolution layer feature maps of immunopositive and immunonegative nuclei patches have been displayed in Fig. 7. The feature maps generated by using various kernels in convolution layers decodes the signature of the expression level of color content of brown (for immunopositive) and blue (for immunonegative) nuclei. In this context, Fig. 7 has been revised by presenting two immunopositive and immunonegative images. It can be observed that the ki-67 expression is different with respect to filters for immunopositive and immunonegative nuclei. Basically from the feature maps, we can assume a nucleus is immunopositive or, not. But for the confirmation, we have to classify the image. Due to a clear visualization of feature maps, only a few feature maps in each layer have been displayed. The qualitative and hotspots detection results have been shown in Figs 8 and 9 respectively. Hence, we can conclude that our proposed methodology is performing better than the existing ones.

### Computational time

Computation time is one of the most vital factors of machine learning. For this reason, we have always tried to keep the patch size as small as possible. The small patch size decreases the computation time and increases the detection performance. The model took almost 5 days (24 × 5 = 120 hours) for training and, on average, takes 1.33 seconds (in GPU) and 1.64 seconds (in CPU) to detect the hotspots (immunopositive and immunonegative nuclei). In comparison, the method in N. Khan *et al*.^31^ requires an average of 7 seconds only to segment a color image. Overall, the proposed method is much more efficient than the existing Ki-67 scoring methods.

### Automated Ki-67 proliferation scoring (APS)

The automated Ki-67 proliferation scoring has been calculated using the below equation^32^

20 $$APS( \% )=\frac{TIP}{TIP+TIN}\times 100$$

here, the TIP = total number of immunopositive nuclei and the TIN = total number of immunonegative nuclei. Table 6 shows the overall proliferation score based on two pathologists and our automated technique. In this table reference range shows the standard proliferated category and their ranges, which are already gold standard in pathology. We compared the proliferation score of both the pathologists’ with the proposed technique. It is observed that in both the cases error rate is negligible. More specifically, in the less proliferated category error rate is 0.06%, average proliferated category error rate is 0.01% and highly proliferated category error rate is 0%. It can be observed that the proposed deep learning framework provides consistent and efficient results as evident from the similar performance.

**Table 6:** Overall proliferation score.

*Reference range* ^26^	*Pathologists*	*MPS (%)*	*APS (%)*
Less proliferate (<15%)	Expert-1	12.87	13.00
	Expert-2	13.01	13.00
	***Average***	***12.94***	***13.00***
Average proliferate (16–30%)	Expert-1	27.29	27.99
	Expert-2	28.00	27.99
	***Average***	***27.64***	***27.99***
Highly proliferate (>31%)	Expert-1	90.00	90.00
	Expert-2	90.00	90.00
	***Average***	***90.00***	***90.00***

*‘MPS’: Manual Proliferation Score*.

**Figure 10:**
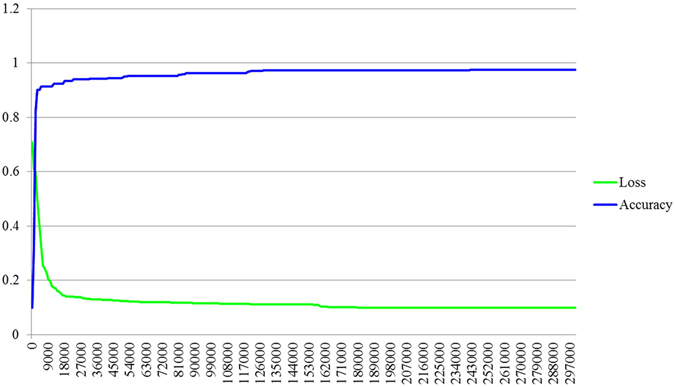
Training performance graph.

**Figure 11:**
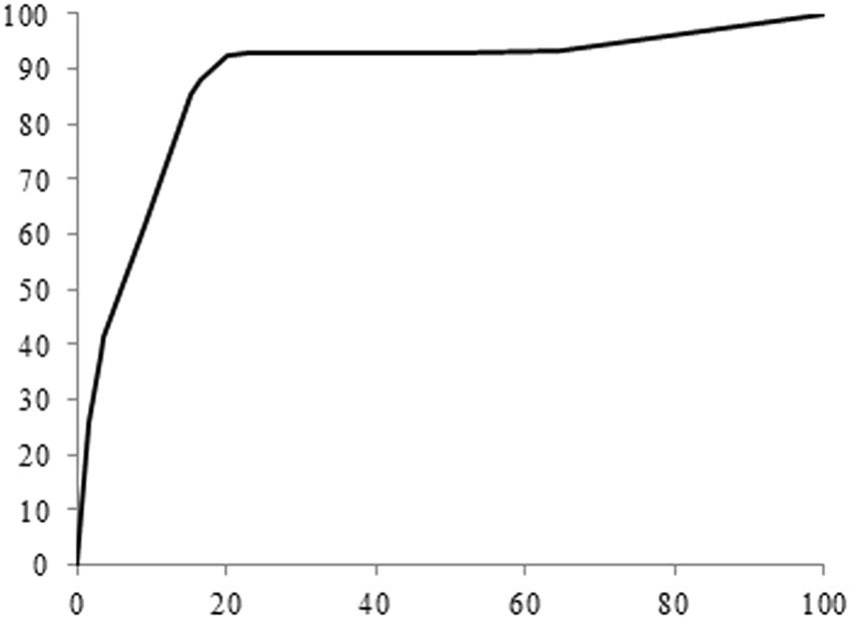
ROC graph for showing the overall performance of the proposed methodology.

The training performance graph has been shown in Fig. 10. After 297,000 iterations the accuracy and loss graph become saturated. Hence we have only shown the graph up to 297,000 iterations. Figure 11 illustrates the ROC curve and the area under the curve (AUC) is 91.

### Comparison with the existing methods

From the exhaustive literature review, it is evident that the quantification and proliferation rate scoring of Ki-67 stained BC or other cancer images using deep learning approach has not been attempted so far. Moreover, there have few limitations, e.g., nonstandard dataset, conventional imaging approach, etc. for which we cannot directly measure the performances of our proposed method with the existing methodologies. Based on some technical understanding of image similarities, we compared the qualitative and quantitative performances with the two recently published articles^15, 31^ on Ki-67 scoring in Table 7. Furthermore, we measured the efficiency of our proposed framework with other conventional methods. Table 8 shows the comparison of performance measures with various combinations, e.g. proposed method but without decision layer, GMM and random forest but without deep network, GMM plus SVM but without deep network, replacing the decision layer with additional FC layer and the proposed methodology. From the Table 8 it is obvious that proposed method including decision layer provides better performance in terms of precision, recall and F-sore value in comparison with the other techniques. Henceforth, the proposed methodology is far better than the existing methods.

**Table 7:** Comparison with the existing methods.

Comparison parameters	P. Shi *et al*.^15^	N. Khan *et al*.^31^	Proposed Methodology
Image type	Human nasopharyngeal carcinoma Xenografts	Neuroendocrine tumor	Breast cancer
Sample size	100 images	57 images	450 images
Image size	2040 × 1536	10 × 5 K	2048 × 1536
Image Magnification	40x	40x	40x
Methodology used	Conventional techniques (smoothing, color channel decomposition, local feature extraction, K-means, watershed segmentation)	Conventional technique (Perceptual clustering)	**Deep Learning integrated with decision layer**
Accuracy (%)	91.8	94.60	**97**
Computation time (sec)	1.7	7	**1.33 in GPU and 1.64 in CPU**
CPU or GPU used	CPU	CPU	**CPU and GPU both**
Error rate	0.82	Not mentioned	**0.41**

**Table 8:** Comparison of performance measures.

Conditions	Pr (%)	Re (%)	F-score (%)
*Without decision layer*	89	80	82
*GMM* + *random forest*	**91**	65	76
*GMM + SVM*	93	80	86
*Replacing decision layer with additional FC layer*	87	**88**	87
*Proposed methodology*	**93**	**88**	**91**

## Conclusion

In this manuscript, our contribution is twofold, (*i*) development of an efficient deep learning model comprises of decision layer for automated detection of hotspots, and (*ii*) development of an automatic proliferation rate scoring technique of Ki-67 positively stained BC images. The proposed deep learning model is capable of computing the scoring index with any IHC image, provided that immunopositive nuclei will manifest as brown color and immunonegative nuclei will show as blue color. The proposed framework starts with a seed point detection using GMM which makes the algorithm more robust. This step substantially eliminates unnecessary background objects. Our proposed deep learning model considers both, the pathologist’s information as well as spatial similarity while detecting hotspots. Our quantitative and qualitative evaluation results showed the better performance of our proposed model. The model provides higher learning accuracy and performance scores as measured by precision, recall and F-score, in comparison with the existing conventional techniques for Ki-67 scoring. The model performance has also been compared with the pathologists’ manual annotations. Prospectively, this model will be highly beneficial to the pathologists for fast and efficient Ki-67 scoring from breast IHC (cancer) images.

## Electronic supplementary material

Supplementary source code and installation guide

Supplementary source code
