# Supplementary material for: An Advanced Deep Learning Approach for Ki-67 Stained Hotspot Detection and Proliferation Rate Scoring for Prognostic Evaluation of Breast Cancer
Source: Sci Rep. 2017 Jun 12;7:3213. doi: 10.1038/s41598-017-03405-5 (PMC5468356; doi:10.1038/s41598-017-03405-5)
Supplement: Supplementary file 1 — Supplementary source code and installation guide [file 41598_2017_3405_MOESM1_ESM.doc]

**An Advanced Deep Learning Approach for Ki-67**

**Stained Hotspot Detection and Proliferation Rate**

**Scoring for Prognostic Evaluation of Breast Cancer**

**Monjoy Saha**^1^**, Chandan Chakraborty**^1,*^**, Indu Arun**^2^**, Rosina Ahmed**^2^**, and Sanjoy Chatterjee**^2^

^1^School of Medical Science and Technology, Indian Institute of Technology, Kharagpur, West Bengal, India

^2^Tata Medical Center, New Town, Kolkata, West Bengal, India

[**Corresponding author’s email:* chandanc@smst.iitkgp.ernet.in](mailto:*Corresponding author's email: chandanc@smst.iitkgp.ernet.in)

**Supplementary materials (source code)**

**CAFFE installation and compilation procedure**

The installation and compilation process of our source codes are similar with official CAFFE installation and compilation process (<http://caffe.berkeleyvision.org/installation.html>). Here, we have explained and summarized the whole process in a very simpler way for the first time user. Our CAFFE source code supports both CPU and GPU. As a supplementary material we are only sharing source codes for CPU.

**A. INSTALLATION GUIDELINES**

**1. Prerequisites**

The below softwares and hardwares are the minimum requirement for successful installation and compilation of CAFFE. We have tested on Ubuntu 14.04 operating system.

***Operating system:*** Ubuntu 14.04

***Software:*** CUDA, BLAS, BOOST >=1.55, protobuf, glog, gflags, hdf5, cuDNN (for GPU acceleration), Matlab, Python, lmdb, leveldb

***Hardware:*** NVIDIA Titan X pascal GPU, AMD Opteron processor 128 GB RAM

**2. Supportive software installation code**

Open terminal and run the following codes. These are the supportive softwares which are necessary for the successful compilation of CAFFE

sudo apt-get install libprotobuf-dev libleveldb-dev libsnappy-dev libopencv-dev libhdf5-serial-dev protobuf-compiler

sudo apt-get install --no-install-recommends libboost-all-dev

sudo apt-get install libgflags-dev libgoogle-glog-dev liblmdb-dev

**3. Compilation**

Configure the Makefile.config file as per the system configuration.

Here, we have given configured Makefile.config as per our system details. Path of the Makefile.config is

$CAFFEROOT/caffe/Makefile.config

run the following codes one by one in the terminal for successful compilation of CAFFE

make all

make test

make runtest

For interfacing/ wrapping with MATLAB and Python run make matcaffe and make pycaffe respectively.

**B. SOURCE CODES**

***Training and testing codes are in the below folders (folder names are marks as bold)***

1.$CAFFEROOT/caffe/examples/ki_67_scoring/**train_val_ki67_09_11_2016.prototxt**

2.$CAFFEROOT/caffe/examples/ki_67_scoring/**deploy_image.prototxt**

3.$CAFFEROOT/caffe/examples/ki_67_scoring/**solver.prototxt**

***Decision layer Codes (for CPU only)***

***1. Class declaration of the proposed decision layer has been kept in the below folder***

$CAFFEROOT/caffe/include/caffe/layers/**decision_layer.hpp**

The name of the file is “**decision_layer.hpp”**

***2. Implementation of decision layer has been placed at***

$CAFFEROOT/caffe/src/caffe/layers/ **decision_layer.cpp**

The name of the file is “**decision_layer.cpp**”

Additional information:

- No backward computation is required
- Available layer-specific ID is 42 where decision layer has been Instantiate and registered.

References from main manuscript:

34. Jia, Y. *et al*. Caffe: Convolutional architecture for fast feature embedding. In Proceedings of the 22nd ACM international conference on Multimedia, 675–678 (ACM, 2014).

42. <https://github.com/bonz0/Decision-Tree>, access on March 31, 2017
